# Family Dysfunctional Interactive Patterns and Alexithymia in Adolescent Patients with Restrictive Eating Disorders

**DOI:** 10.3390/children9071038

**Published:** 2022-07-12

**Authors:** Chiara Coci, Livio Provenzi, Valentina De Giorgis, Renato Borgatti, Matteo Chiappedi, Martina Maria Mensi, on behalf of the Mondino Foundation Eating Disorders Clinical and Research Group

**Affiliations:** 1 Department of Brain and Behavioral Sciences, University of Pavia, 27100 Pavia, Italy; chiara.coci01@universitadipavia.it (C.C.); livio.provenzi@mondino.it (L.P.); valentina.degiorgis@mondino.it (V.D.G.); renato.borgatti@mondino.it (R.B.); martina.mensi@mondino.it (M.M.M.); 2 Child Neurology and Psychiatry Unit, IRCCS Mondino Foundation, 27100 Pavia, Italy; 3 Vigevano Child Neurology and Psychiatry Unit, ASST Pavia, 27100 Pavia, Italy

**Keywords:** alexithymia, adolescence, anorexia nervosa, family functioning, Lausanne Trilogue Play, restrictive eating disorders

## Abstract

Adolescents diagnosed with Restrictive Eating Disorders (REDs) are at risk for alexithymia. REDs patients’ families show dysfunctional interactive patterns, and childhood family environment influences alexithymia development. We aimed to assess the relationship between family dysfunctional interactive patterns and patients’ alexithymia in a sample of adolescents diagnosed with REDs. Forty-five patients and their parents were enrolled. They participated in the clinical version of the Lausanne Triadic Play (LTPc), a standardized observational procedure to assess family functioning. We used the self-report questionnaire Toronto Alexithymia Scale (TAS-20) to assess patients’ alexithymia. The TAS-20 provides a multi-factorial measure of patients’ alexithymia: Difficulty in Identifying Feelings, DIF; Difficulty in Describing Feelings, DDF; Externally-oriented Thinking, EOT) and a total (TOT) score. DDF and EOT scores were significantly higher than DIF score. Patients’ families showed dysfunctional interactive patterns, with a predominance of collusive alliance. Patients from families characterized by collusive alliance had higher TOT scores compared to counterparts from families exhibiting a different interactive dysfunctional pattern. In families characterized by a collusive triadic alliance, the dysfunctional interactive pattern was linked with the risk of alexithymia in patients with REDs. Assessment of family relationships should be included in the routine consultation with adolescent patients affected by REDs.

## 1. Introduction

Restrictive eating disorders (REDs) are a heterogeneous group of psychopathological conditions characterized by restricted oral intake associated with moderate to high levels of psychosocial and work impairment [1,2]. REDs’ etiology is multi-factorial, depending on specific biological, psychological, environmental, familiar, socio-cultural risk factors, combined with an individual vulnerability, mainly expressed during adolescence [2,3,4,5,6,7]. It is necessary to look at these disorders with a broad and comprehensive view that considers the many variables involved in the development and maintenance of this complex disorder. Previous evidence indicates that adolescent patients diagnosed with REDs show a broad range of emotional dysfunctions, including poor emotion recognition and deficits in emotional information processing, a psychological feature called alexithymia [8,9,10]. This cognitive-affective deficit is characterized by three main dimensions: difficulty in identifying feelings, difficulty in describing feelings, and externally-oriented thinking. Difficulty in identifying feelings includes struggling in distinguishing emotions from physical sensations and being confused about which emotions are experienced. Difficulty in describing feelings is about struggling in sharing and describing emotional states to others. Finally, externally-oriented thinking concerns the preference to let things happen rather than reflect on their causes [11,12,13]. Alexithymia is identified as a personality trait that predisposes to the development of psychopathology [14], and previous studies have shown a clear prevalence in patients with eating disorders compared to the healthy population [10]. Alexithymia appears to be involved in the onset, maintenance, and even response to treatment, thus making it an important focus in patient care and therapeutic intake [15,16].

Moreover, dysfunctional interactive patterns have been described in REDs adolescents’ families, identified especially in the maintenance mechanism [2,17,18,19]. The presence of dysfunctional interactive patterns can be assessed by using the clinical version of the Lausanne Trilogue Play (LTPc), a validated observational procedure that allows the analysis of families’ triadic interactions [20]. In the LTPc, families are asked to take part in a videotaped four-phase semi-structured interactive game that replicates their everyday real-life functioning. By observing the quality of family members’ interactive coordination, it is possible to characterize the type of family alliance. By using this instrument, previous studies have shown that families of adolescents with REDs present a deteriorated interaction quality, especially in the triadic phase, where families were required to act with greater coordination [21,22,23]. Moreover, Mensi and colleagues identified a distinctive REDs profile of family functioning, characterized by a collusive alliance in most of the families [21,24]. In other words, the various members of these families struggle to support each other to achieve the goal of the game, because of poor coordination [25].

Both alexithymia and dysfunctional interactional family patterns in adolescents have been independently correlated with REDs. At the same time, previous studies have identified a significant correlation between alexithymia in psychiatric patients and their parental bonding styles, supporting the idea that alexithymia may turn out to be the developmental response to a specific parenting style [26]. Despite this the relationship between each other have not been explored extensively, Mannarini and Kleinbub recently conducted a study assessing alexithymia and parental bonding in 32 Italian families consisting of an adolescent with anorexia, a sibling, and their parents [27]. They identified higher levels of alexithymia in patients than in other family members. Higher levels of alexithymia were also related to neglectful parenting styles [27]. Relying on what is known in the literature, we hypothesized that, in adolescents with REDs, high levels of alexithymia are associated with the presence of collusive alliance in their families. Indeed, the aim of the present study was to determine if there is a significant relationship between family dysfunctional interactive patterns and patients’ alexithymia in a sample of adolescents diagnosed with REDs.

## 2. Materials and Methods

### 2.1. Participants

**Figure 1:**
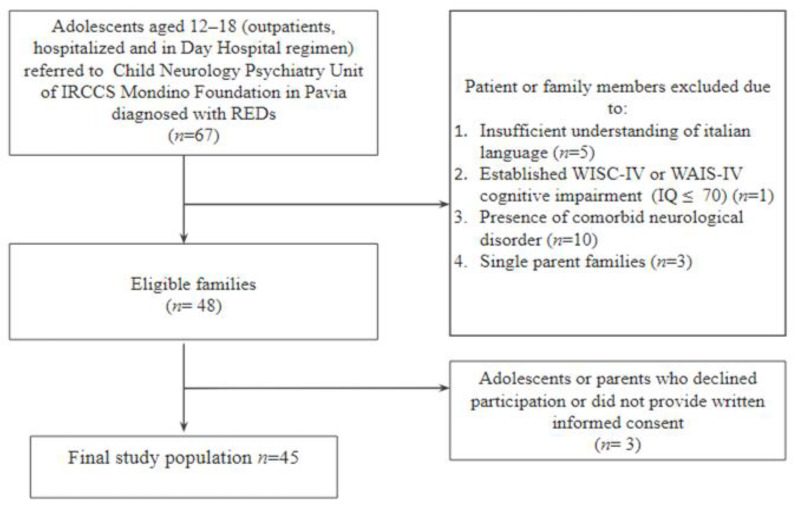
Flowchart of study population. IRCCS: Istituto di Ricovero e Cura a Carattere Scientifico (scientific hospitalization and treatment institute). REDs: Restrictive Eating Disorders.

We enrolled 45 adolescent patients diagnosed with REDs during hospitalization at the Child Neurology and Psychiatry Unit of IRCCS Mondino Foundation in Pavia. All participants were Caucasian and lived in Italy. We included patients aged 12 to 18 years, with a diagnosis of REDs, including Anorexia Nervosa, ARFID, and Anorexia NAS according to the Diagnostic and Statistical Manual of Mental Disorders (DSM-5) criteria [28]. Adolescents were not eligible for the study if they presented any comorbid neurological disorder, intellectual disability or autism spectrum disorder, according to DSM-5 criteria, before the enrolment. The same exclusion criteria were also applied to parents. Furthermore, we excluded participants who had an insufficient understanding of the Italian language (Figure 1). To confirm appropriate RED diagnosis and exclude the presence of comorbidities, patients and their caregivers were interviewed using the DSM-based Kiddie Schedule for Affective Disorders and Schizophrenia (K-SADS) [29]. The Structured Clinical Interview for DSM-5 Personality Disorders (SCID-5 PD) [30] was administered to the patients to evaluate the presence of any personality disorders. The absence of intellectual disability was assessed using the appropriate Wechsler intelligence scale according to patients’ age [31,32].

All enrolled patients and their parents provided written informed consent to participate in the study. The study received the approval of the Ethics Committee of Policlinico San Matteo in Pavia (Protocol ID: P-20170016006). The authors assert that all procedures contributing to this work comply with the ethical standards of the relevant national and institutional committees on human experimentation and with the Helsinki Declaration of 1964 and its latter amendments. The dataset is available upon reasonable request to the corresponding author and it will be accessible through the Open Science online Zenodo repository.

### 2.2. Procedures

A trained child neuropsychiatrist interviewed parents and patients to collect socio-demographic and clinical data and performed a comprehensive clinical examination including medical and family history, and neurological and psychiatric assessments. Adolescent patients were asked to complete the self-report Toronto Alexithymia Scale (TAS-20) questionnaire [33,34], a validated scale for the measure of alexithymia [35]. To evaluate the presence of family dysfunctional interactive patterns, patients took part in the LTPc [20] with their parents. Every session was videotaped in a dedicated room, and subsequently coded off-line by two independent trained judges.

### 2.3. Measures

*Toronto Alexithymia Scale (TAS-20)* [33,36]

Each statement of TAS-20 is rated on a Likert scale from 1 (complete disagreement) to 5 (complete agreement). A total score (TOT) is obtained by summing the item ratings; a score between 61 and 100 indicates clinical alexithymia risk. Three subscales are also provided: difficulty in identifying feelings (DIF), difficulty in describing feelings (DDF), and externally-oriented thought (EOT), i.e., a tendency to focus on external events rather than their own internal affective state. Subscales do not present a clinical cut-off; however, higher scores reflect worse functioning in the specific subdomains of alexithymia.

*Lausanne Trilogue Play—clinical version (LTPc)* [20]

LTPc is a standardized and well-validated procedure to assess family functioning. The game is structured in four phases: in three phases, two members act together while the third one just observes, without taking part in the interaction (Phase I and Phase II: one parent plays with the patient and the other one assumes the role of observer; Phase IV: parents talk together, while the adolescent finishes the task independently), and in one phase the three members interact together (Phase III). The LTPc coding allows describing the interactive contribution of each family member and the overall family functioning through four functional level codes (i.e., participation, organization, focalization, affective contact) for each phase. A total family score ranging from 0 to 40 is obtained, identifying four types of family alliance: cooperative, in tension, collusive and disturbed [25]. Cooperative alliances (32–40) identify families that cooperate as a team, creating a positive atmosphere, and promoting the child’s adaptation. Similarly, tension alliance (24–31) identifies a functional family model, although these families show greater difficulties in correcting each other’s errors. In collusive alliances, (16–23) major complications in the parental system emerge: affective climate is constantly in tension and both parents and child struggle to support each other to achieve the goal, due to difficult coordination between the family members. Finally, in disturbed alliances (0–15) families are unable to complete the task due to the loss of family balance, the roles are confused, or one member is excluded from the triad.

### 2.4. Statistical Analysis

To assess the presence of significant differences among the TAS subscales (i.e., DIF, DDF, and EOT), a within-subject analysis of variance (ANOVA) was used on the entire sample of patients. Repeated contrasts were planned to test for pairwise mean differences in the case of a significant ANOVA test. As the collusive alliance has been previously described in families of adolescents with a diagnosis of RED, family dysfunctional interactive pattern was recoded dichotomously as collusive alliance (1 = yes, 0 = no). To test for the presence of a statistically significant difference in TAS TOT or in the TAS subscales (i.e., DIF, DDF, EOT) between triads divided by collusive alliance or non-collusive alliances, separate t-tests for independent samples were used. The analyses were conducted using SPSS 27 for Windows. A two-tailed α < 0.05 criterion for statistical significance was adopted for all the analyses.

## 3. Results

Table 1 shows the descriptive statistics and clinical characteristics of the sample. At the study enrollment, patients had received individual psychotherapy, *n* = 24 (53%), family psychotherapy, *n* = 2 (4%), or parental couple psychotherapy, *n* = 6 (13%). Five families (11%) had received more than one intervention concurrently. While all the subjects received a diagnosis of RED, 21 (47%) also presented comorbidity for a depressive disorder, whereas other comorbidities included anxiety disorder, *n* =4 (9%), obsessive-compulsive disorder, *n* = 3 (7%), personality disorder, *n* = 2 (4%), bipolar disorder, *n* = 2 (4%), and schizophrenia, *n* = 1 (2%).

**Figure 2:**
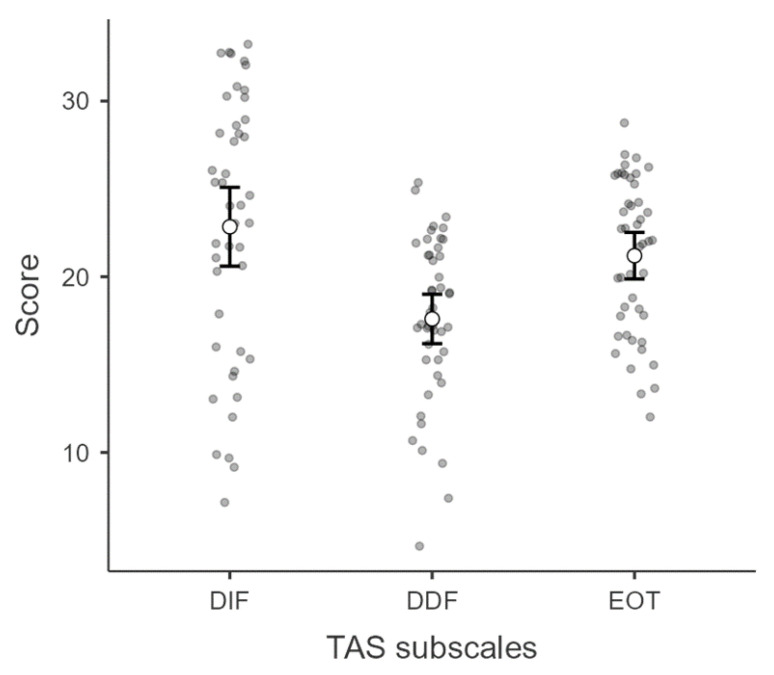
Distribution of TAS subscales scores in our sample. DIF, Difficulty Identifying Feelings; DDF, Difficulty Describing Feelings; EOT, Externally-Oriented Thinking.

In the whole sample, a significant difference emerged for TAS subscales, F(2,88) = 18.95, *p* < 0.001, η^2^ = 0.30: RED patients reported lower scores for the DDF subscale compared to DIF, t(44) = 6.66, *p* < 0.001, and EOT, t(44) = 5.21, *p* < 0.001 (Figure 2).

Collusive alliance was the prevalent dysfunctional interactive pattern among the families included in the study (n = 18, 40%), followed by stressed alliance (n = 14, 31%) and disordered alliance (n = 13, 29%). No families showed a cooperative alliance. A more comprehensive table including descriptive statistics for the sample by family alliance style is available in Supplementary Tables.

**Figure 3:**
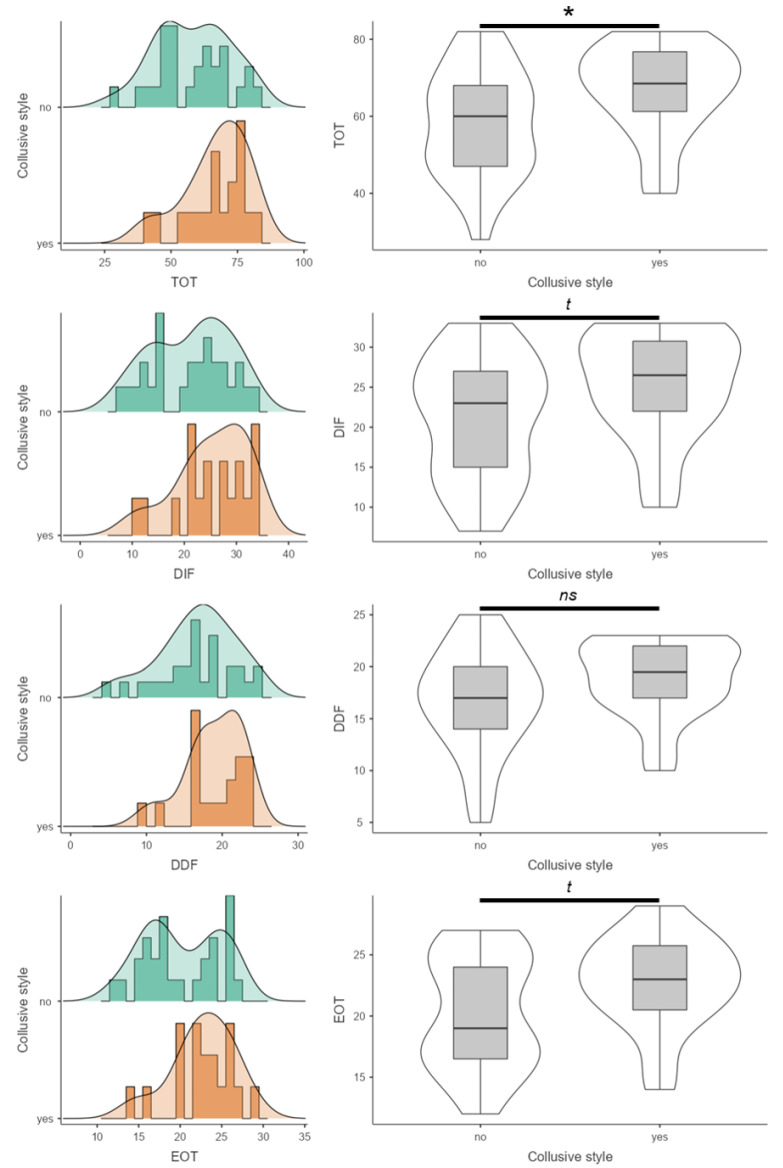
Comparison of TAS scores between collusive and non-collusive families. * = *p* < 0.05; t = tendency to statistical significance; ns = statistically not significant. DIF, Difficulty Identifying Feelings; DDF, Difficulty Describing Feelings; EOT, Externally-Oriented Thinking; TOT, Total Score.

No differences emerged for socio-demographic characteristics and clinical variables by collusive alliance. Table 1 also reports the mean scores and standard errors for TAS TOT and subscales’ scores for both groups, collusive alliance and other alliances. Adolescents with a family rated as collusive reported higher TOT scores compared to counterparts from families who did not show a collusive alliance, t(43) = 1.88, *p* = 0.033, η^2^ = 0.10. Moreover, statistical trends emerged for specific TAS subscales: adolescents from collusive families tended to report higher scores in DIF, t(43) = 1.88, *p* = 0.067, η^2^ = 0.08, and EOT, t(43) = 1.95, *p* = 0.058, η^2^ = 0.08, compared to patients from non-collusive ones (Figure 3).

## 4. Discussion

The aim of the current study was to explore the association between family dysfunctional interactive patterns and alexithymia in adolescent patients with REDs. Our results suggested that there may be a significant association between higher levels of alexithymia and the presence of family collusive alliance in adolescents diagnosed with REDs.

We found that, in our patients’ sample, two specific subscales of alexithymia—DIF and EOT—showed higher scores when compared to the third DDF dimension. As shown by the effect size (i.e., η^2^p = 0.30) this difference was statistically robust. The presence of difficulties in recognizing emotions, and the tendency to adopt an externally-oriented style of thinking, could significantly interfere with patients’ ability to recognize and express their inner suffering, leading to greater psychosomatic reactions. This finding confirms previous studies that highlighted how patients suffering from REDs may find specific difficulties in identifying their own and others’ emotional states [10,12]. On the other hand, previous evidence of an association between EOT and REDs is mixed [12,37,38,39,40]. It is possible to speculate that while a specific difficulty in identifying feelings may be a core feature of alexithymia in these patients, the presence of an externally-oriented thinking style should not be considered as a feature or stable trait, but rather a potentially dysfunctional aspect that may be observed only in individual patients. We could hypothesize that environmental factors—e.g., the presence of specific dysfunctional interactive patterns in the family functioning—may facilitate the emergence of specific alexithymia features in these patients. Nonetheless, due to the cross-sectional nature of the present study, it cannot be excluded that in families of adolescents with REDs the risk of developing a collusive alliance pattern increases. Therefore, to better comprehend the causative association between patients’ risk for alexithymia and family dysfunctional interactive patterns, future longitudinal studies are needed.

Furthermore, none of the families enrolled in this study exhibited an optimal interactive pattern, whereas a collusive alliance emerged as the prevalent dysfunctional interactive pattern in 40% of the sample. This finding confirms previous reports: Balottin and colleagues [21] reported a similar prevalence of the collusive alliance pattern in 12 out of 20 families of adolescent patients with REDs. Moreover, the present result further highlights that collusive alliance may be a common family dysfunctional pattern in adolescents diagnosed with REDs. In these families, parents are frequently unable to ensure proper guidance to their children by failing to respect proposed roles, and children seem to struggle to reach an independent role and to mature their own personal ideas [21,22].

Finally, we compared the levels of alexithymia recorded on the TAS scale between the group of families characterized by a collusive alliance, recognized in the literature as prevalent in REDs, and those that are not collusive. Thus, exploring the association between family functioning and patient’s alexithymia, we were able to observe the presence of a significant correlation: adolescents from families exhibiting a collusive alliance had higher levels of alexithymia TOT score and the effect was medium (η^2^ = 0.10). This kind of family alliance is characterized by difficulties in adhering to the assigned role during the LTPc procedure. Unlike the disordered alliance, a family structure is usually preserved, despite there often being significant open or hidden competition for control between parents. Within this interactive family style, it is easy to hypothesize that affective experiences are often poorly understood and only partially shared among family members, hindering the patient’s development of emotional sensitivity, and contributing to the emergence of alexithymia traits. On the other hand, avoidance of emotional exploration and sharing inevitably aggravates family conflicts.

Moreover, exploring the TAS subscales, we found that adolescents from collusive families also showed a tendency to report greater DIF and EOT scores compared to families who did not exhibit the same interactive pattern. Notably, these are the subscales that emerged as specifically problematic in these patients. Despite this association not reaching statistical significance, the presence of a statistical tendency may suggest that these specific subscales or subdomains of the alexithymia construct should be specifically explored in future studies on the interactive patterns of families of adolescents with REDs.

A limitation of this study is the relatively small sample size. Of course, this was due to the need to include only families in which both parents were available to take part in the LTPc procedure. This limits the generalization of the present results and future replications are needed in larger samples. Nonetheless, the availability of observational measures of family interactive patterns is a strength of this study. Additionally, alexithymia was assessed with a self-report tool, and it is not free from the biases associated with self-reported measures. The lack of a comparison group with families of healthy adolescents is another limitation.

## 5. Conclusions

Families of adolescent patients diagnosed with REDs usually develop specific dysfunctional interactive patterns characterized by a collusive alliance. At the same time, these patients often manifest high levels of alexithymia with specific difficulties in identifying feelings and, in our sample, exhibiting externally-oriented thinking. Notably, this study further adds that there may be a significant association between the adolescent patients’ risk for alexithymia and the presence of a specific pattern of family interactive dysfunction, namely collusive alliance. By highlighting a specific link between features of family functioning and the affective wellbeing of adolescent patients, these findings have critical implications for clinical practice. The detection of alexithymic traits and the study of family dynamics can support the clinician in the early identification of individuals predisposed to the development of REDs and thus enable the initiation of prompt and accurate treatment. Several studies suggest prioritizing a family-centered approach to the assessment and treatment of REDs [2,26,41,42]. By including a systematic evaluation of family interactive patterns in clinical practice, clinicians may identify relational risk factors linked with greater maladaptive emotional functioning, such as alexithymia. The clinical practice with adolescent patients with REDs may thus benefit from the assessment of both family functioning and alexithymia, promoting a family-centered approach in psychiatric settings.

## Figures and Tables

**Table 1:** Descriptive statistics for the whole sample and split by collusive alliance.

				Collusive Alliance			
		All (*N* = 45)		Yes (*N* = 18)		No (*N* = 27)	
		***N***	**%**	***N***	**%**	***N***	**%**
Sex	Males	9	20.0	3	16.7	6	22.2
	Females	36	80.0	15	83.3	21	77.8
Socially withdrawn	No	40	88.9	16	88.9	24	88.9
	Yes	5	11.9	2	11.9	3	11.9
Academic retreat	No	37	82.2	12	66.7	25	92.6
	Yes	8	17.8	6	33.3	2	7.4
Self-harm behaviors	No	36	80.0	13	72.2	23	85.2
	Yes	9	20.0	5	27.8	4	14.8
Comorbid affective symptoms	No	24	53.3	8	44.4	16	59.3
	Yes	21	46.7	10	55.6	11	40.7
		**Mean**	**SD**	**Mean**	**SD**	**Mean**	**SD**
Demographic characteristics	Patients’ age (years)	14.91	1.59	14.78	1.63	15.11	1.57
	Patients’ weight (kg)	40.20	7.35	39.53	7.37	31.22	7.40
	Patients’ BMI (kg/m^2^)	15.88	2.50	16.52	2.33	15.45	2.57
	Fathers’ age (years)	51.00	5.53	51.91	5.78	49.76	5.08
	Mothers’ age (years)	48.25	5.41	48.43	5.69	48.00	5.16
TAS scores	DIF	22.84	7.47	25.33	6.82	21.19	7.54
	DDF	17.60	4.68	18.89	3.76	16.74	5.08
	EOT	21.20	4.41	22.72	3.79	20.19	4.57
	TOT	61.64	13.74	66.94	11.91	58.11	13.94

Abbreviations: BMI, Body Mass Index; TAS, Toronto Alexithymia Scale; DIF, Difficulty Identifying Feelings; DDF, Difficulty Describing Feelings; EOT, Externally-Oriented Thinking; TOT, Total score.

## Data Availability

Data are available upon reasonable request from Zenodo (http:10.5281/zenodo.49432).

## Supplementary Materials

The following supporting information can be downloaded at: https://www.mdpi.com/article/10.3390/children9071038/s1, Tables with descriptive statistics for the sample by family alliance style.
