# Supplementary material for: Family Dysfunctional Interactive Patterns and Alexithymia in Adolescent Patients with Restrictive Eating Disorders
Source: Children (Basel). 2022 Jul 12;9(7):1038. doi: 10.3390/children9071038 (PMC9323591; doi:10.3390/children9071038)
Supplement: Supplementary file 1 [file children-09-01038-s001.zip › children-1773992-supplementary.pdf]

Supplementary Materials: Tables with Descriptive statistics for the sample by family alliance style (disorganized, n = 13; collusive, n = 18; tense, n = 14).

Frequencies of Sex

	Family alliance style		
**Sex**	**disorganized**	**collusive**	**tense**
Female	10	15	11
Male	3	3	3

Frequencies of Social relations

	Family alliance style		
**Social relations**	**disorganized**	**collusive**	**tense**
social retirement	2	2	1
poor	4	9	5
adequate	7	7	7

Frequencies of Academic functioning

	Family alliance style		
**Academic functioning**	**disorganized**	**collusive**	**tense**
retirement	1	6	1
poor	1	0	0
sufficient	4	2	1
good	3	7	5
optimal	4	3	7

Frequencies of Risky behaviors

	Family alliance style		
**Risky behaviors**	**disorganized**	**collusive**	**tense**
no	10	13	13
self-harm	2	4	1
substance abuse	1	0	0
suicidal attempts	0	1	0

Frequencies of Comorbidity

	Family alliance style		
Comorbidity	**disorganized**	**collusive**	**tense**
no	8	8	8
yes	5	10	6

	**Family alliance style**	**Mean**	**SD**
Patients’ age (years)	disorganized	14.9	1.32
	collusive	15.1	1.57
	tense	14.6	1.91
Patients‘ weight (Kg)	disorganized	41.2	9.58
	collusive	41.2	7.40
	tense	37.9	4.28
Patients’ BMI (Kg/m^2)	disorganized	16.0	3.54
	collusive	16.5	2.33
	tense	14.9	1.01
Fathers’ age (years)	disorganized	53.5	5.84
	collusive	49.8	5.08
	tense	50.5	5.58
Mothers’ age (years)	disorganized	49.9	6.02
	collusive	48.0	5.16

Frequencies of Comorbidity

	Family alliance style			
Comorbidity	**disorganized**	**collusive**	**tense**	
	tense		47.1	5.25

	**Family alliance style**	**Mean**	**SD**
DIF	disorganized	22.2	7.17
	collusive	25.3	6.82
	tense	20.3	8.02
DDF	disorganized	17.3	4.79
	collusive	18.9	3.76
	tense	16.2	5.48
EOT	disorganized	20.2	4.97
	collusive	22.7	3.79
	tense	20.1	4.37
TOT	disorganized	59.7	13.22
	collusive	66.9	11.91
	tense	56.6	14.92
